# Step up to triple therapy versus switch to dual bronchodilator therapy in patients with COPD on an inhaled corticosteroid/long-acting β_2_-agonist: post-hoc analyses of KRONOS

**DOI:** 10.1186/s12931-025-03234-5

**Published:** 2025-05-08

**Authors:** Dave Singh, Mona Bafadhel, Niki Arya, Jonathan Marshall, Himanshu Parikh, Dobrawa Kisielewicz, Charlotta Movitz, Karin Bowen, Mehul Patel

**Affiliations:** 1 Medicines Evaluation Unit, University of Manchester, Manchester University NHS Foundation Hospitals Trust, Manchester, M23 9QZ UK; 2 King’s Centre for Lung Health, School of Immunology and Microbial Sciences, Faculty of Life Science and Medicine, King’s College London, London, UK; 3 Respiratory and Immunology, Biometrics, BioPharmaceuticals R&D, AstraZeneca, Durham, NC USA; 4 Global Medical Affairs - Respiratory, BioPharmaceuticals Medical, AstraZeneca, Cambridge, UK; 5 Respiratory and Immunology, Clinical Development, BioPharmaceuticals R&D, AstraZeneca, Gaithersburg, MD USA; 6 Respiratory and Immunology, Clinical Development, Biopharmaceuticals R&D, AstraZeneca, Barcelona, Spain; 7 Respiratory and Immunology, Biometrics, BioPharmaceuticals R&D, AstraZeneca, Gothenburg, Sweden; 8 Respiratory and Immunology, Biometrics, BioPharmaceuticals R&D, AstraZeneca, Gaithersburg, MD USA; 9 Respiratory and Immunology, Clinical Development, BioPharmaceuticals R&D, AstraZeneca, Cambridge, UK

**Keywords:** Chronic obstructive pulmonary disease, Exacerbations, Lung function, Triple therapy

## Abstract

**Background:**

In people with chronic obstructive pulmonary disease (COPD) on inhaled corticosteroid/long-acting β_2_-agonist (ICS/LABA) therapy, the Global Initiative for Chronic Obstructive Lung Disease (GOLD) recommends stepping up to ICS/long-acting muscarinic antagonist (LAMA)/long-acting β2-agonist (LABA) in those with exacerbations or switching to LAMA/LABA in those with major symptoms. However, the effect of stepping up to ICS/LAMA/LABA versus switching to LAMA/LABA on exacerbation risk is unclear. This analysis evaluated the effect of escalating to ICS/LAMA/LABA versus switching to LAMA/LABA or staying on ICS/LABA on lung function and exacerbation rates in symptomatic individuals with COPD without a recent exacerbation history from KRONOS.

**Methods:**

In KRONOS (NCT02497001), symptomatic participants with moderate-to-very severe COPD (exacerbations in the prior year were not required for inclusion) were randomized to budesonide/glycopyrronium/formoterol fumarate dihydrate 320/14.4/10 μg (BGF), glycopyrronium/formoterol fumarate dihydrate 14.4/10 μg (GFF), budesonide/formoterol fumarate dihydrate 320/10 μg (BFF) via metered-dose inhaler, or budesonide/formoterol fumarate dihydrate 400/12 μg via dry-powder inhaler (BUD/FORM) for 24 weeks. In participants without a recent exacerbation history on ICS/LABA in the 30 days before screening, morning pre-dose trough FEV_1_ change from baseline and moderate/severe exacerbation rates over 24 weeks were analyzed post-hoc using linear repeated measures models and negative binomial regression, respectively, and participants escalated to ICS/LAMA/LABA (BGF) were compared with those switching to LAMA/LABA (GFF) or staying on ICS/LABA (BFF or BUD/FORM).

**Results:**

On stepping up to BGF, least square means (95% confidence interval [CI]) differences for morning pre-dose trough FEV_1_ change from baseline over 24 weeks was similar versus switching to GFF (12 [–21, 44] mL) but greater versus staying on ICS/LABA (BGF vs. BFF, 106 [64, 148] mL; BGF vs. BUD/FORM, 55 [12, 97] mL). Moderate/severe exacerbations were experienced by participants in all treatment arms (BGF, 14.9%; GFF, 24.0%; BFF 17.6%; BUD/FORM, 21.2%). Exacerbation risk was reduced when stepping up to BGF versus switching to GFF (rate ratio [95% CI]: 0.57 [0.35, 0.94]); rate ratios (95% CI) for BGF versus remaining on ICS/LABA were 0.93 (0.47, 1.82) with BFF and 0.62 (0.33, 1.18) with BUD/FORM.

**Conclusions:**

People with symptomatic COPD and no recent exacerbation history previously on ICS/LABA had reduced exacerbation risk after escalating to ICS/LAMA/LABA versus switching to LAMA/LABA, and improved lung function versus staying on ICS/LABA.

**Trial registration:**

ClinicalTrials.gov registry number NCT02497001 (registration date, 7 July 2015).

**Supplementary Information:**

The online version contains supplementary material available at 10.1186/s12931-025-03234-5.

## Background

Inhaled corticosteroid (ICS)/long-acting β_2_-agonist (LABA) combination therapy has been used as a maintenance treatment for chronic obstructive pulmonary disease (COPD) for many years [1, 2]. A substantial proportion of people living with COPD remain on ICS/LABA, with the real-world EXACOS international study of patients living with COPD in low- and middle-income countries reporting ICS/LABA single inhalers being prescribed in > 40% of the study population [3]. However, the 2023 and 2024 Global Initiative for Chronic Obstructive Lung Disease (GOLD) reports no longer recommend the use of ICS/LABA for the maintenance of COPD, stating that “*Use of LABA + ICS is not encouraged. If there is an indication for ICS*,* then LABA + LAMA + ICS has been shown to be superior to LABA + ICS and is therefore the preferred choice*” [4, 5]. The IMPACT and ETHOS studies that underpin this GOLD recommendation both showed that triple therapy with ICS/long-acting muscarinic antagonist (LAMA)/LABA is superior to dual therapy on a range of endpoints [6, 7].

For people living with COPD receiving dual ICS/LABA therapy, GOLD 2025 recommends that treatment should be stepped up to triple therapy with ICS/LAMA/LABA (i) in those with current exacerbations and a blood eosinophil count ≥ 100 cells/mm^3^ or (ii) in those with no current exacerbations and a previous positive response to ICS/LABA but with high symptom burden [8]. A switch to LAMA/LABA should be considered in those with (i) current exacerbations and blood eosinophil count < 100 cells/mm^3^, or (ii) no relevant exacerbation history documented in their medical records [8]. However, individuals who experience no, or few, exacerbations of COPD can still exhibit a high symptom burden (such as chronic cough or sputum production) or have low lung function [9, 10]. Increased breathlessness and frequent productive cough (cough and sputum production most/several days per week in past 3 months) are also associated with increased exacerbation risk [11, 12]. Similarly, there can still be a risk of exacerbations in individuals with physician-assigned COPD of mild severity, or in symptomatic patients at low exacerbation risk [13, 14].

The Phase III KRONOS study (NCT02497001), which included a majority of participants (74%, *n* = 1411/1896) who had not experienced an exacerbation in the prior year while either receiving dual or triple inhaled therapies, reported that budesonide/glycopyrronium/formoterol fumarate dihydrate (BGF) 320/14.4/10 μg (equivalent to budesonide/glycopyrrolate/formoterol fumarate 320/18/9.6 μg) improved lung function and reduced exacerbation rates versus dual LAMA/LABA therapy with glycopyrronium/formoterol fumarate dihydrate 14.4/10 μg (GFF) and versus dual ICS/LABA therapy with budesonide/formoterol fumarate 320/10 μg (BFF) via metered-dose inhaler (MDI) or budesonide/formoterol fumarate dihydrate 400/12 μg via dry-powder inhaler (DPI; BUD/FORM) [15]. Similarly, BGF reduced moderate or severe exacerbation rates and improved lung function versus dual LAMA/LABA and ICS/LABA therapies in the 52-week Phase III ETHOS study [6].

Evidence is currently limited as to whether a step up to ICS/LAMA/LABA from ICS/LABA confers lower exacerbation risk versus a switch to LAMA/LABA from ICS/LABA in patients without a recent exacerbation history. Therefore, further examination of clinical outcomes in patients after such changes is warranted. The primary aim of the current post-hoc analysis was to evaluate lung function and exacerbation rates in the subpopulation of KRONOS study participants without a history of exacerbations in the previous 12 months who were receiving ICS/LABA in the 30 days before screening, after escalation to ICS/LAMA/LABA with BGF versus: switching to LAMA/LABA with GFF ***or*** staying on ICS/LABA with BFF or BUD/FORM. To supplement these analyses, additional analyses of lung function and exacerbation rates were also conducted in the following populations: (i) the overall population on ICS/LABA in the 30 days before screening (regardless of exacerbation history in the past 12 months) and (ii) participants on ICS/LABA in the 30 days before screening with no exacerbation history in the previous 12 months who had moderate COPD.

## Methods

### Study design

The full details of the KRONOS study design have been published previously [15]. Briefly, KRONOS (NCT02497001) was a double-blind, parallel-group, multicentre, multinational (Canada, China, Japan, and the USA) Phase III randomized study in symptomatic participants with moderate-to-very severe COPD. Eligible participants were randomized 2:2:1:1 to BGF 320/14.4/10 μg, GFF 14.4/10 μg, BFF 320/10 μg via MDI, or open-label BUD/FORM 400/12 μg via DPI (Symbicort Turbuhaler®; equivalent to an estimated delivered dose of 320/9 μg) as two twice-daily inhalations for 24 weeks. All participants received salbutamol sulphate as rescue medication (as needed) throughout the study.

The KRONOS study was conducted in accordance with Good Clinical Practice, including the Declaration of Helsinki. The study protocol and informed consent forms were approved by appropriate institutional review boards or independent ethics committees (see [15] for a complete listing). All patients provided written informed consent before screening.

### Participants

Detailed inclusion and exclusion criteria have been previously reported [15]. In brief, eligible participants were aged 40–80 years, were current or former smokers, had an established clinical history of COPD, and had moderate-to-very severe COPD (defined by a postbronchodilator forced expiratory volume in 1 s [FEV_1_] < 80% and ≥ 25% predicted normal). Participants were required to be symptomatic, as indicated by a COPD Assessment Test score ≥ 10, despite receiving ≥ 2 inhaled maintenance therapies for ≥ 6 weeks before screening, but were not required to have a COPD exacerbation within the preceding year. Participants were excluded if they had a current diagnosis of asthma or any respiratory disease other than COPD that could affect study results, and if they exhibited an acute worsening of COPD requiring oral corticosteroids or antibiotics within 6 weeks of screening or during screening.

### Outcomes

Prespecified primary and secondary endpoints from the KRONOS study have been previously reported [15]. A primary endpoint in the KRONOS study was change from baseline in morning pre-dose trough FEV_1_ over 24 weeks.

The rate of moderate/severe COPD exacerbations was a secondary endpoint. Exacerbations were defined as changes in the usual COPD symptoms lasting ≥ 2 days that were beyond normal day-to-day variation and acute in onset, and possibly warranting a change in regular medication. Exacerbations included ≥ 1 major symptom (dyspnea, sputum volume, or sputum color) and ≥ 1 other major or minor symptom (cough, wheeze, sore throat, cold symptoms [rhinorrhea or nasal congestion], or fever without other cause). Moderate exacerbations were those requiring systemic corticosteroid or antibiotic treatment (or both) for ≥ 3 days, or ≥ 1 depot injectable dose of corticosteroids. Severe exacerbations were those leading to hospitalization or a healthcare facility visit (e.g., emergency department) lasting ≥ 24 h or COPD-related death.

In the current post-hoc analyses, lung function (morning pre-dose trough FEV_1_ change from baseline) and moderate/severe exacerbation rates over 24 weeks were analysed based on inhaled maintenance therapy combination subgroup (ICS/LABA) during the 30 days before screening. The timepoint of ‘over 24 weeks’ was chosen so data from each scheduled assessment contributed to the endpoint. As a result, there is less impact of missing data at a single scheduled assessment and, consequently, variability is decreased. The main analysis examined treatment effects among participants without a history of exacerbations in the previous 12 months who were on ICS/LABA in the 30 days before screening from the modified intention-to-treat (mITT) population, hereafter referred to as the ‘Exacerbations^(No recent)^’ population. Analyses focused on comparisons between those who stepped up to ICS/LAMA/LABA triple therapy with BGF versus those who switched to LAMA/LABA with GFF (BGF versus GFF) and those who remained on ICS/LABA with BFF or BUD/FORM (BGF versus BFF and BGF versus BUD/FORM). Scheduled (non-pro re nata) short-acting β_2_-agonists (SABA) and/or short-acting muscarinic antagonists (SAMA) were considered maintenance therapies, and so could be included in ‘LAMA’ or ‘LABA’ use.

To supplement these analyses, comparable examinations of treatment effects were conducted in participants on ICS/LABA in the 30 days before screening from the overall mITT population (i.e., regardless of exacerbation history), and among those without a history of exacerbations who had moderate COPD (defined as FEV_1_ of 50-80% predicted normal) from the mITT population, hereafter referred to as the ‘Exacerbations^(No recent + moderate COPD)^’ population.

### Statistical analysis

All analyses were conducted in the mITT population (participants randomized to treatment who received any amount of study drug, and who had post-randomization data obtained prior to discontinuation from treatment). Demographic and clinical characteristics are reported using descriptive statistics. Change from baseline in morning pre-dose trough FEV_1_ was assessed using linear repeated measures models, which included baseline FEV_1_, percent reversibility to salbutamol sulphate and baseline eosinophil count as continuous covariates and visit, treatment and the treatment by visit interaction as categorical covariates.

For assessment of exacerbation rates, negative binomial regression was used to generate rate ratios (RRs) and corresponding 95% confidence intervals (CIs). The model was adjusted for baseline post-bronchodilator percent predicted FEV_1_ and baseline eosinophil count as continuous covariates and country as a categorical covariate, with baseline COPD exacerbation history (0, 1, > 2) also included as a categorical covariate in analyses of the overall population of participants regardless of exacerbation history. The time at risk of experiencing an exacerbation was used as an offset variable in the model. Exacerbations were considered separate events provided ≥ 7 days occurred between the stop date of the earlier event and start date of the later. The annual exacerbation rate was calculated as the total number of exacerbations divided by the total years of exposure across all patients for the treatment. Time during an exacerbation or in the 7 days following an exacerbation was not included in the calculation of exposure.

The KRONOS study was not prospectively powered for any of the reported post-hoc analyses.

## Results

### Participant disposition and demographics

As previously reported [15], of 1902 randomized participants in KRONOS, 1896 were included in the mITT population. Of the 1411 patients from the Exacerbations^(No recent)^ population, 554 (39%) were on ICS/LABA in the 30 days before screening.

Baseline demographics and clinical characteristics were similar across treatment arms among participants in the Exacerbations^(No recent)^ population (Table 1). Similar patterns were observed for those on ICS/LABA in the 30 days before screening in the overall mITT, i.e., regardless of exacerbation history in the prior 12 months (Supplementary Table S1 in Additional file 1) and among those in the Exacerbations^(No recent + moderate COPD)^ population (Supplementary Table S2 in Additional file 1).

**Table 1:** Baseline characteristics: participants in the Exacerbations^(No recent)^ population^a^

	Step up to ICS/LAMA/LABA	Switch to LAMA/LABA	Stay on ICS/LABA	
	BGF 320/14.4/10 μg (*N* = 188)	GFF 14.4/10 μg (*N* = 196)	BFF 320/10 μg (*N* = 85)	BUD/FORM 400/12 μg (*N* = 85)
Age,^b^ mean years (SD)	63.7 (7.5)	63.3 (7.9)	64.3 (6.7)	63.6 (7.2)
Sex, n (%)				
Female	56 (29.8)	65 (33.2)	23 (27.1)	26 (30.6)
Male	132 (70.2)	131 (66.8)	62 (72.9)	59 (69.4)
Current smoker, n (%)	84 (44.7)	93 (47.4)	34 (40.0)	39 (45.9)
Moderate or severe exacerbations in the previous year, n (%)				
None (0)	188 (100.0)	196 (100.0)	85 (100)	85 (100)
Exacerbation number (past 12 months), mean (SD)	NA	NA	NA	NA
Blood eosinophil count > 100 cells/mm^3^, n (%)	137 (72.9)	142 (72.4)	59 (69.4)	55 (64.7)
Blood eosinophil count ≥ 150 cells/mm^3^, n (%)	98 (52.1)	101 (51.5)	46 (54.1)	29 (34.1)
Post-bronchodilator FEV_1_% predicted, mean (SD)	51.7 (14.1)	51.7 (13.9)	49.6 (11.9)	51.6 (13.9)
Reversible to bronchodilator,^c^ n (%)	91 (48.4)	97 (49.5)	42 (49.4)	40 (47.1)
Total CAT score, mean (SD)	19.0 (6.8)	18.9 (6.5)	19.5 (7.3)	19.2 (6.6)

^a^Participants on ICS/LABA in the 30 days before screening and without exacerbation history in the previous 12 months. ^b^The age at the time of informed consent. ^c^Reversibility was defined as an improvement in FEV_1_ after salbutamol administration (compared with before salbutamol administration) of 12% or more and 200 mL or more

BFF, budesonide/formoterol fumarate dihydrate (via MDI); BGF, budesonide/glycopyrronium/formoterol fumarate dihydrate; BUD/FORM, budesonide/formoterol fumarate dihydrate (via DPI); CAT, COPD Assessment Test; COPD, chronic obstructive pulmonary disease; DPI, dry-powder inhaler; FEV_1_, forced expiratory volume in 1 s; GFF, glycopyrronium/formoterol fumarate dihydrate; ICS, inhaled corticosteroid; LABA, long-acting β_2_-agonist; LAMA, long-acting muscarinic antagonist; MDI, metered-dose inhaler;NA, not applicable; SD, standard deviation

### Treatment effects on lung function

**Fig. 1:**
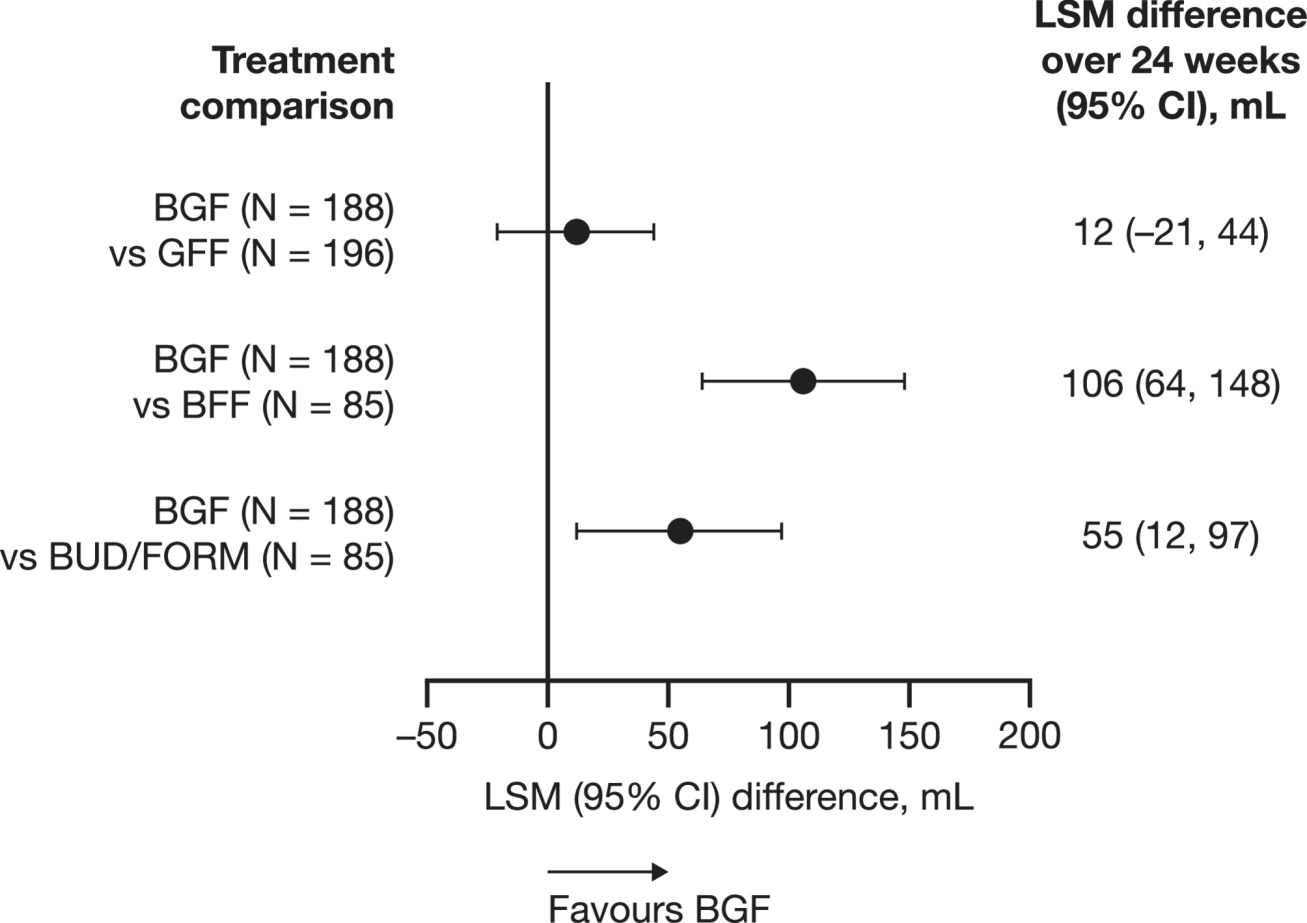
Lung function change from baseline^a^: participants in the Exacerbations^(No recent)^ population^b^ ^a^Baseline was defined as the mean of all evaluable 60- and 30-minute morning pre-dose values on Day 1 (Visit 4) and LSM are presented. ^b^Participants on ICS/LABA in the 30 days before screening and without exacerbation history in the previous 12 months. BFF, budesonide/formoterol fumarate dihydrate (via MDI); BGF, budesonide/glycopyrronium/formoterol fumarate dihydrate; BUD/FORM, budesonide/formoterol fumarate dihydrate (via DPI); CI, confidence interval; DPI, dry-powder inhaler; GFF, glycopyrronium/formoterol fumarate dihydrate; ICS, inhaled corticosteroid; LABA, long-acting β_2_-agonist; LSM, least squares mean; MDI, metered-dose inhaler

Among participants in the Exacerbations^(No recent)^ population, morning pre-dose trough FEV_1_ at baseline was comparable across treatment groups (Table 2), with the same pattern observed in the overall mITT population and the Exacerbations^(No recent + moderate COPD)^ population (Supplementary Table S3 in Additional file 1). The least squares mean (LSM) difference in morning pre-dose trough FEV_1_ change from baseline over 24 weeks was similar for those who stepped up to BGF versus those who switched to LAMA/LABA with GFF (LSM [95% CI] difference, 12 [–21, 44] mL), but was greater when stepping up to BGF versus staying on ICS/LABA with BFF (LSM [95% CI] difference, 106 [64, 148] mL) or BUD/FORM (LSM [95% CI] difference, 55 [12, 97] mL) (Fig. 1). Findings were similar for participants in the overall mITT population (Supplementary Figure S1a in Additional file 1). In those from the Exacerbations^(No recent + moderate COPD)^ population, morning pre-dose trough FEV_1_ change from baseline over 24 weeks was only greater when stepping up to BGF versus staying on ICS/LABA with BFF (Supplementary Figure S1b in Additional file 1).

**Table 2:** Lung function changes from baseline^a^: participants in the Exacerbations^(No recent)^ population^b^

	Step up to ICS/LAMA/LABA	Switch to LAMA/LABA	Stay on ICS/LABA	
	BGF 320/14.4/10 μg	GFF 14.4/10 μg	BFF 320/10 μg	BUD/FORM 400/12 μg
*n*	188	188	83	79
Mean morning pre-dose trough FEV_1_ ± SD at baseline, L	1.242 ± 0.479	1.248 ± 0.482	1.159 ± 0.393	1.209 ± 0.504
LSM (SE) change in morning pre-dose trough FEV_1_ from baseline over 24 weeks, L	0.149 (0.0117)	0.137 (0.0118)	0.043 (0.0177)	0.094 (0.0181)
(95% CI), L	(0.126; 0.172)	(0.114; 0.160)	(0.008; 0.077)	(0.058; 0.129)

^a^Baseline was defined as the mean of all evaluable 60- and 30-minute pre-dose values on Day 1 (Visit 4). ^b^Participants on ICS/LABA in the 30 days before screening and without exacerbation history in the previous 12 months

BFF, budesonide/formoterol fumarate dihydrate (via MDI); BGF, budesonide/glycopyrronium/formoterol fumarate dihydrate; BUD/FORM, budesonide/formoterol fumarate dihydrate (via DPI); CI, confidence interval; DPI, dry-powder inhaler; FEV_1_, forced expiratory volume in 1 s; GFF, glycopyrronium/formoterol fumarate dihydrate; ICS, inhaled corticosteroid; LABA, long-acting β_2_-agonist; LAMA, long-acting muscarinic antagonist; LSM, least squares mean; MDI, metered-dose inhaler; SE, standard error; SD, standard deviation

### Treatment effects on exacerbation rates

**Fig. 2:**
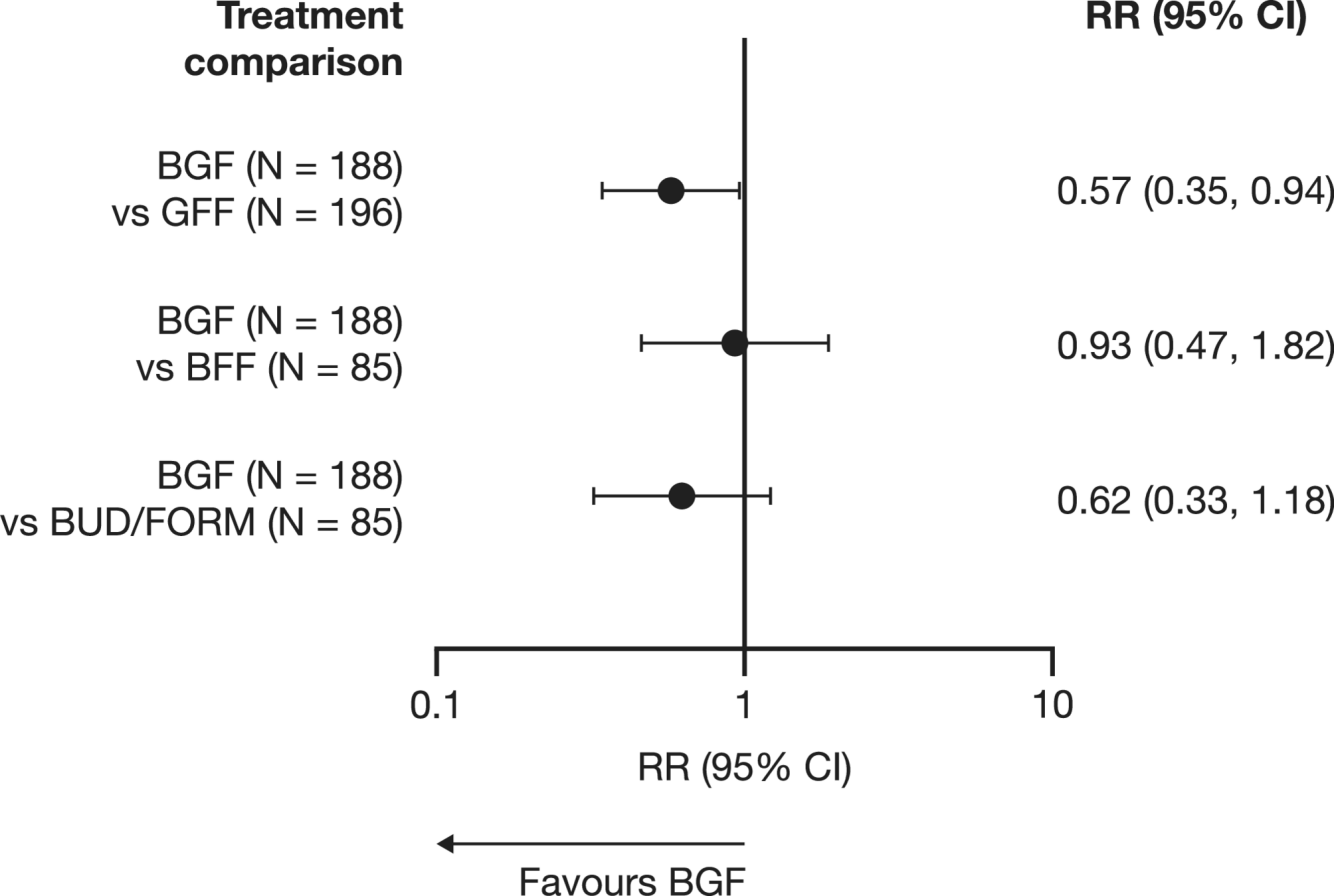
Moderate/severe COPD exacerbation rate ratios: participants in the Exacerbations^(No recent)^ population^a^ ^a^Participants on ICS/LABA in the 30 days before screening and without exacerbation history in the previous 12 months. BFF, budesonide/formoterol fumarate dihydrate (via MDI); BGF, budesonide/glycopyrronium/formoterol fumarate dihydrate; BUD/FORM, budesonide/formoterol fumarate dihydrate (via DPI); CI, confidence interval; COPD, chronic obstructive pulmonary disease; DPI, dry-powder inhaler; GFF, glycopyrronium/formoterol fumarate dihydrate; ICS, inhaled corticosteroid; LABA, long-acting β_2_-agonist; MDI, metered-dose inhaler; RR, rate ratio

Annual rates of moderate/severe exacerbations among participants in the Exacerbations^(No recent)^ population are reported in Table 3. The rate of moderate/severe exacerbations was reduced by 43% among participants who stepped up to BGF versus those who switched to LAMA/LABA with GFF, with an observed RR (95% CI) of 0.57 (0.35, 0.94). When stepping up to BGF versus staying on ICS/LABA, RRs (95% CI) were 0.93 (0.47, 1.82) with BFF and 0.62 (0.33, 1.18) with BUD/FORM (Fig. 2).

**Table 3:** Moderate/severe exacerbation rates: participants in the Exacerbations^(No recent)^ population^a^

	Step up to ICS/LAMA/LABA	Switch to LAMA/LABA	Stay on ICS/LABA	
	BGF 320/14.4/10 μg	GFF 14.4/10 μg	BFF 320/10 μg	BUD/FORM 400/12 μg
*n*	188	196	85	85
Patients with exacerbations, n (%)	28 (14.9)	47 (24.0)	15 (17.6)	18 (21.2)
Events, n	36	59	17	22
Estimated rate of exacerbations (SE) over 24 weeks, per year^b, c^	0.43 (0.08)	0.76 (0.13)	0.47 (0.13)	0.70 (0.18)

COPD exacerbations were considered separate events provided that 7 or more days were between the recorded stop date of the earlier event and start date of the later

^a^Participants on ICS/LABA in the 30 days before screening and without exacerbation history in the previous 12 months. ^b^Model-estimated rate. ^c^The rate of exacerbations per year was the total number of exacerbations divided by the total years of exposure across all participants for the treatment. Time during an exacerbation or in the 7 days following an exacerbation was not included in the calculation of exposure

BFF, budesonide/formoterol fumarate dihydrate (via MDI); BGF, budesonide/glycopyrronium/formoterol fumarate dihydrate; BUD/FORM, budesonide/formoterol fumarate dihydrate (via DPI); COPD, chronic obstructive pulmonary disease; DPI, dry-powder inhaler; GFF, glycopyrronium/formoterol fumarate dihydrate; ICS, inhaled corticosteroid; LABA, long-acting β_2_-agonist; LAMA, long-acting muscarinic antagonist; MDI, metered-dose inhaler; SE, standard error

Similar patterns were observed for the supportive analyses (Supplementary Table S4 and Supplemental Figure S2 in Additional file 1). Among those in the overall mITT population, the rate of moderate/severe exacerbations was 54% lower when stepping up to BGF versus switching to LAMA/LABA with GFF. RRs (95% CI) for treatment differences in exacerbation rates for stepping up to BGF versus staying on ICS/LABA were 0.73 (0.43, 1.26) with BFF and 0.65 (0.39, 1.08) with BUD/FORM (Supplementary Figure S2a in Additional file 1).

Among those in the Exacerbations^(No recent + moderate COPD)^ population, the rate of moderate/severe exacerbations was 68% lower among those who stepped up to BGF versus those who switched to GFF. Treatment differences in exacerbation rates for stepping up to BGF versus staying on ICS/LABA were 0.72 (0.27, 1.96) with BFF and 0.64 (0.25, 1.67) with BUD/FORM (Supplementary Figure S2b in Additional file 1).

## Discussion

This post-hoc analysis of the KRONOS study analysed lung function and moderate/severe exacerbation rates among those on ICS/LABA during the 30 days prior to screening, with a focus on those participants without an exacerbation history within the previous year (i.e., the Exacerbations^(No recent)^ population); a visual summary of this analysis can be found in the graphic abstract in Additional file 2. We observed that among participants in the mITT without a recent exacerbation history 39% were being treated with ICS/LABA in the 30 days before screening. Escalation from ICS/LABA to ICS/LAMA/LABA provided a lung function benefit that was similar compared to switching to dual therapy with LAMA/LABA, but greater compared with continuing dual therapy with ICS/LABA. Furthermore, exacerbation risk was reduced when stepping up to BGF versus switching to LAMA/LABA.

The greater improvement in lung function with ICS/LAMA/LABA and LAMA/LABA compared with continuing ICS/LABA was expected since a bronchodilator was added with ICS/LAMA/LABA and LAMA/LABA treatment. However, exacerbation risk was substantially reduced with BGF versus LAMA/LABA. Additionally, the estimated exacerbation rate among participants who switched to GFF (0.76) was not reduced versus those who stayed on ICS/LABA with BUD/FORM (0.70) or BFF (0.47), indicating that a switch to LAMA/LABA does not provide an exacerbation benefit versus staying on ICS/LABA.

In additional analyses of participants on ICS/LABA in the 30 days before screening in the overall mITT population (regardless of exacerbation history) and among those in the Exacerbations^(No recent + moderate COPD)^ population, similar findings were observed.

Taken together, these findings indicate that escalation to triple therapy from ICS/LABA dual therapy provides benefits on lung function and exacerbation risk, although the nature of the benefit differs according to the comparator (ICS/LABA or LAMA/LABA). The observation that similar results are observed even in the in the Exacerbation^(No recent + moderate COPD)^ population is important because it indicates the benefits of stepping up to ICS/LAMA/LABA are not driven only by participants with severe or very severe disease or those with an exacerbation history in the previous year. These results may support earlier intervention approaches with triple therapy given the treatment effect observed with BGF among patients who are symptomatic, with no recent exacerbations and without severe lung function impairment.

GOLD 2025 recommends a switch to LAMA/LABA in individuals with COPD currently on ICS/LABA who have no features of asthma and have no relevant exacerbation history documented in their medical records [8]. This scenario differs from individuals who had previous exacerbations but who have responded positively to ICS/LABA treatment and now have no recent exacerbations; these individuals may escalate to triple therapy on the basis of symptom burden. The KRONOS study (and hence the current post hoc analysis) was not able to distinguish these two subgroups, as close inspection of the medical records for each individual is required. Nevertheless, data from these post-hoc analyses provide support for GOLD 2025 recommendations in patients with no current exacerbations but high symptom load, suggesting an exacerbation benefit (i.e. reduced risk) is observed from a step up to triple therapy versus a switch to LAMA/LABA in individuals with no exacerbations in the prior year.

The participants included in this analysis were considered stable from the perspective of exacerbations, but were experiencing a substantive symptom burden, as indicated by CAT scores ≥ 10. In COPD, disease stability is a multifactorial concept in which exacerbations should be considered along with quality of life, lung function and symptoms [16]. Previous work has shown that individuals exhibiting symptoms of COPD including dyspnea [17] and frequent productive cough [12], are at increased risk of exacerbations. As such, it is important to consider the burden of COPD beyond a history of exacerbations and the risk they pose for future exacerbations, as further exacerbations can be associated with irreversible lung function decline, risk of cardiovascular events and mortality [18–23]. Taken together, these findings suggest stepping up to ICS/LAMA/LABA may be the preferred treatment option in those on ICS/LABA, as opposed to a switch to LAMA/LABA. Although the current analyses did not include an examination of safety/adverse events (AE), a previously published sub-analysis of KRONOS showed that treatment-emergent AE profiles were generally comparable across treatment arms in patients with and without exacerbations in the 12 months prior to the study [24]. Furthermore, the incidence of confirmed pneumonia was low across treatment arms for both subgroups [24].

While many of the study participants included in this analysis did not experience an exacerbation in 12 months prior to screening, it is possible those on ICS/LABA prior to screening had a past history of exacerbations (beyond 12 months) and that their physician may have maintained ICS-based treatment due to this exacerbation history.

A limitation of this post-hoc analysis is that the study was not powered to demonstrate treatment differences in these endpoints for these subpopulations. Furthermore, due to the small sample sizes in the ICS/LABA subgroups (i.e., BUD/FORM and BFF) and the overlapping confidence intervals for comparisons with BGF, caution should be taken when drawing conclusions regarding the comparative efficacy of these treatments. It should also be noted that the 24-week observation period may be considered a relatively short time period to adequately assess treatment effects on exacerbation rates. In addition, it has been noted that the removal of ICS in COPD can result in worsening of disease [25, 26]. In the current analyses, this potential ‘withdrawal effect’ could impact those participants on ICS/LABA in the 30 days before screening who switched to LAMA/LABA. However, previous analyses based on the ETHOS study have indicated that ICS withdrawal does not appear to account for the benefits of BGF [27]. Finally, it should be noted that the effects observed in this report may not be generalizable to ICS/LAMA/LABA types that differ to those used in the current analysis.

## Conclusions

The proactive management of exacerbation risk in patients who remain symptomatic on ICS/LABA should include considering the potential benefits of a step up to ICS/LAMA/LABA compared with a switch to LAMA/LABA, based on the reduced risk of exacerbations demonstrated herein. These data may help inform the management of patients who are symptomatic with no recent exacerbations who are on ICS/LABA therapy, including those with moderate COPD. Other datasets, especially from populations not restricted to requiring exacerbations or significant FEV_1_ limitation, could be used to help inform clinical practice and guidelines.

## Electronic supplementary material

Below is the link to the electronic supplementary material.

Supplementary Material 1: Additional file 1: The supplementary material includes 2 supplementary Tables, and 2 supplementary figures with data from the overall mITT population, and participants without a recent exacerbation, and moderate COPD

Supplementary Material 2: Additional file 2: This document contains a visual summary of the study, including key takeaway points from the background, methods, results and conclusions

## Data Availability

Data underlying the findings described in this manuscript may be obtained in accordance with AstraZeneca’s data sharing policy described at https://astrazenecagrouptrials.pharmacm.com/ST/Submission/Disclosure. Data for studies directly listed on Vivli can be requested through Vivli at www.vivli.org. Data for studies not listed on Vivli could be requested through Vivli at https://vivli.org/members/enquiries-about-studies-not-listed-on-the-vivli-platform/. The AstraZeneca Vivli member page is also available outlining further details: https://vivli.org/ourmember/astrazeneca/.

## Declarations

### Prior publication

These data have been presented in part at the 2023 European Respiratory Society International Congress, (Milan, Italy; 9–13 September 2023) as a poster, with the associated published abstract (Eur Respir 62: PA4687; 10.1183/13993003.congress-2023.PA4687).

## Abbreviations

AE: Adverse event
BFF: Budesonide/formoterol fumarate dihydrate
BGF: Budesonide/glycopyrronium/formoterol fumarate dihydrate
BUD/FORM: Budesonide/formoterol fumarate dihydrate
CI: Confidence interval
COPD: Chronic obstructive pulmonary disease
DPI: Dry-powder inhaler
FEV_1_: Forced expiratory volume in 1 s
GFF: Glycopyrronium/formoterol fumarate dihydrate
GOLD: Global Initiative for Chronic Obstructive Lung Disease
ICS: Inhaled corticosteroid
LABA: Long-acting β_2_-agonist
LAMA: Long-acting muscarinic antagonist
LSM: Least squares means
MDI: Metered-dose inhaler
mITT: Modified intention-to-treat
RR: Rate ratio
SABA: Short-acting β_2_-agonist
SAMA: Short-acting muscarinic antagonist
