# Supplementary material for: Step up to triple therapy versus switch to dual bronchodilator therapy in patients with COPD on an inhaled corticosteroid/long-acting β2-agonist: post-hoc analyses of KRONOS
Source: Respir Res. 2025 May 8;26:175. doi: 10.1186/s12931-025-03234-5 (PMC12063277; doi:10.1186/s12931-025-03234-5)
Supplement: Supplementary file 1 — Supplementary Material 1: Additional file 1: The supplementary material includes 2 supplementary Tables, and 2 supplementary figures with data from the overall mITT population, and participants without a recent exacerbation, and moderate COPD [file 12931_2025_3234_MOESM1_ESM.docx]

# Supplementary information

**Step up to triple therapy versus switch to dual bronchodilator therapy in patients with COPD on an inhaled corticosteroid/long-acting β2-agonist: post-hoc analyses of KRONOS**

Dave Singh^1^, Mona Bafadhel^2^, Niki Arya^3^, Jonathan Marshall^4^, Himanshu Parikh^5^, Dobrawa Kisielewicz^6^, Charlotta Movitz^7^, Karin Bowen^8^ and Mehul Patel^9^

*^1^Medicines Evaluation Unit, University of Manchester, Manchester University NHS Foundation Hospitals Trust, Manchester, UK;* *^2^King’s Centre for Lung Health, School of Immunology and Microbial Sciences, Faculty of Life Science and Medicine, King’s College London, London, UK; ^3^Late Respiratory and Immunology Biometrics, BioPharmaceuticals R&D, AstraZeneca, Durham, NC, USA; ^4^Global Medical Affairs - Respiratory, BioPharmaceuticals Medical, AstraZeneca, Cambridge, UK; ^5^Late Respiratory and Immunology Clinical Development, BioPharmaceuticals R&D, AstraZeneca, Gaithersburg, MD, USA; ^6^Late Respiratory and Immunology Clinical Development, Biopharmaceuticals R&D, AstraZeneca, Barcelona, Spain; ^7^Late Respiratory and Immunology Biometrics, BioPharmaceuticals R&D, AstraZeneca Gothenburg, Sweden; ^8^Late Respiratory and Immunology Biometrics, BioPharmaceuticals R&D, AstraZeneca, Gaithersburg, MD, USA; ^9^Late Respiratory and Immunology Clinical Development, BioPharmaceuticals R&D, AstraZeneca, Cambridge, UK*

**Corresponding author:** Dave Singh. E-mail: [dsingh@meu.org.uk](mailto:dsingh@meu.org.uk)

**Supplementary Table S1**. Baseline characteristics: participants on ICS/LABA before screening,^a^ overall mITT population^b^

|  | **Step up to  ICS/LAMA/LABA** | **Switch to  LAMA/LABA** | **Stay on**  **ICS/LABA** | |
| --- | --- | --- | --- | --- |
|  | **BGF  320/14.4/10 µg (N = 252)** | **GFF  14.4/10 µg (N = 254)** | **BFF  320/10 µg (N = 107)** | **BUD/FORM  400/12 µg (N = 124)** |
| Age,^c^ mean years (SD) | 63.1 (7.7) | 63.4 (7.9) | 63.9 (7.1) | 63.7 (7.6) |
| Sex, n (%) |  |  |  |  |
| Female | 79 (31.3) | 94 (37.0) | 32 (29.9) | 39 (31.5) |
| Male | 173 (68.7) | 160 (63.0) | 75 (70.1) | 85 (68.5) |
| Current smoker, n (%) | 110 (43.7) | 121 (47.6) | 44 (41.1) | 58 (46.8) |
| Moderate or severe exacerbations in the previous year, n (%) |  |  |  |  |
| None (0) | 188 (74.6) | 196 (77.2) | 85 (79.4) | 85 (68.5) |
| 1 | 46 (18.3) | 46 (18.1) | 15 (14.0) | 31 (25.0) |
| ≥ 2 | 18 (7.1) | 12 (4.7) | 7 (6.5) | 8 (6.5) |
| Exacerbation number (past 12 months), mean (SD) | 0.4 (0.9) | 0.3 (0.7) | 0.3 (0.6) | 0.4 (0.6) |
| Blood eosinophil count > 100 cells/mm^3^, n (%) | 177 (70.2) | 188 (74.0) | 78 (72.9) | 85 (68.5) |
| Blood eosinophil count ≥ 150 cells/mm^3^, n (%) | 125 (49.6) | 135 (53.1) | 58 (54.2) | 54 (43.5) |
| Post-bronchodilator FEV_1_ % predicted, mean (SD) | 51.0 (14.2) | 51.8 (14.2) | 50.2 (12.6) | 50.9 (13.3) |
| Reversible to bronchodilator,^d^ n (%) | 124 (49.2) | 118 (46.5) | 54 (50.5) | 57 (46.0) |
| Total CAT score, mean (SD) | 19.1 (6.7) | 18.8 (6.4) | 19.3 (7.2) | 19.5 (6.5) |

^a^In the 30 days before screening. ^b^Full mITT (regardless of exacerbation history in prior 12 months). ^c^The age at the time of informed consent. ^d^Reversibility was defined as an improvement in FEV_1_ after salbutamol administration (compared with before salbutamol administration) of 12% or more and 200 mL or more.

BFF, budesonide/formoterol fumarate dihydrate (via MDI); BGF, budesonide/glycopyrronium/formoterol fumarate dihydrate; BUD/FORM, budesonide/formoterol fumarate dihydrate (via DPI); CAT, COPD Assessment Test; COPD, chronic obstructive pulmonary disease; DPI, dry-powder inhaler; FEV_1_, forced expiratory volume in 1 second;
GFF, glycopyrronium/formoterol fumarate dihydrate; ICS, inhaled corticosteroid; LABA, long-acting β_2_-agonist;
LAMA, long-acting muscarinic antagonist; MDI, metered-dose; mITT, modified intention-to-treat; SD, standard deviation.

**Supplementary Table S2**. Baseline characteristics: participants in the Exacerbations^(No recent +moderate COPD)^ population^a^

|  | **Step up to  ICS/LAMA/LABA** | **Switch to  LAMA/LABA** | **Stay on**  **ICS/LABA** | |
| --- | --- | --- | --- | --- |
|  | **BGF  320/14.4/10 µg (N = 98)** | **GFF  14.4/10 µg (N = 108)** | **BFF  320/10 µg (N = 40)** | **BUD/FORM  400/12 µg (N = 48)** |
| Age,^b^ mean years (SD) | 63.6 (8.5) | 63.2 (8.2) | 64.7 (7.3) | 64.1 (7.8) |
| Sex, n (%) |  |  |  |  |
| Female | 32 (32.7) | 37 (34.3) | 12 (30.0) | 13 (27.1) |
| Male | 66 (67.3) | 71 (65.7) | 28 (70.0) | 35 (72.9) |
| Current smoker, n (%) | 48 (49.0) | 47 (43.5) | 14 (35.0) | 24 (50.0) |
| Moderate or severe exacerbations in the previous year, n (%) |  |  |  |  |
| None (0) | 98 (100%) | 108 (100%) | 40 (100%) | 48 (100%) |
| Exacerbation number (past 12 months), mean (SD) | NA | NA | NA | NA |
| Blood eosinophil count > 100 cells/mm^3^, n (%) | 79 (80.6) | 86 (79.6) | 27 (67.5) | 32 (66.7) |
| Blood eosinophil count ≥ 150 cells/mm^3^, n (%) | 57 (58.2) | 60 (55.6) | 20 (50.0) | 18 (37.5) |
| Post-bronchodilator FEV_1_ % predicted, mean (SD) | 62.8 (8.0) | 62.2 (8.1) | 60.1 (5.9) | 61.8 (8.0) |
| Reversible to bronchodilator,^c^ n (%) | 49 (50.0) | 57 (52.8) | 22 (55.0) | 24 (50.0) |
| Total CAT score, mean (SD) | 18.8 (7.1) | 18.5 (7.0) | 18.3 (7.5) | 18.6 (6.6) |

^a^Participants without a history of exacerbations in the previous 12 months who were on ICS/LABA in the 30 days before screening from the mITT population who had moderate COPD (defined as FEV_1_ 50%–80% predicted normal).^b^The age at the time of informed consent. ^c^Reversibility was defined as an improvement in FEV_1_ after salbutamol administration (compared with before salbutamol administration) of 12% or more and 200 mL or more.

BFF, budesonide/formoterol fumarate dihydrate (via MDI); BGF, budesonide/glycopyrronium/formoterol fumarate dihydrate; BUD/FORM, budesonide/formoterol fumarate dihydrate (via DPI); CAT, COPD Assessment Test; COPD, chronic obstructive pulmonary disease; DPI, dry-powder inhaler; FEV_1_, forced expiratory volume in 1 second;
GFF, glycopyrronium/formoterol fumarate dihydrate; ICS, inhaled corticosteroid; LABA, long-acting β_2_-agonist;
LAMA, long-acting muscarinic antagonist; MDI, metered-dose inhaler; NA, not applicable; SD, standard deviation.

**Supplementary Table S3**. Lung function: participants on ICS/LABA before screening^a^ by population

|  | **Step up to  ICS/****LAMA/LABA** | **Switch to  LAMA/LABA** | **Stay on ICS/LABA** | |
| --- | --- | --- | --- | --- |
|  | **BGF  320/14.4/10 µg** | **GFF  14.4/10 µg** | **BFF  320/10 µg** | **BUD/FORM  400/12 µg** |
| **Overall mITT** | | | | |
| n | 252 | 244 | 105 | 115 |
| Mean morning pre-dose trough FEV_1_ ± SD at baseline^b^, L | 1.206 ± 0.476 | 1.215 ± 0.465 | 1.153 ± 0.402 | 1.200 ± 0.477 |
| LSM (SE) change in morning pre-dose trough FEV_1_ from baseline^b^ over 24 weeks, L | 0.145 (0.0105) | 0.127 (0.0108) | 0.072 (0.0163) | 0.076 (0.0156) |
| (95% CI), L | (0.124; 0.166) | (0.106; 0.149) | (0.040; 0.104) | (0.046; 0.107) |
| **Exacerbations^(No recent + moderate COPD)^** **population^c^** | | | | |
| N | 98 | 106 | 39 | 43 |
| Mean ± SD morning pre-dose trough FEV_1_ at baseline^b^, L | 1.537 ± 0.429 | 1.513 ± 0.429 | 1.397 ± 0.388 | 1.515 ± 0.469 |
| LSM (SE) change in morning pre-dose trough FEV_1_ from baseline^b^ over 24 weeks, L | 0.143 (0.0168) | 0.136 (0.0163) | 0.049 (0.0269) | 0.092 (0.0253) |
| (95% CI), L | (0.110; 0.176) | (0.104; 0.168) | (–0.004; 0.102) | (0.043; 0.142) |

^a^In the 30 days before screening.  ^b^Baseline was defined as the mean of all evaluable 60- and 30-minute pre-dose values on Day 1 (Visit 4) and LSM are presented. ^c^Participants from the mITT population without a history of exacerbations in the previous 12 months who had moderate COPD (defined as FEV_1_ 50%–80% predicted normal).

BFF, budesonide/formoterol fumarate dihydrate (via MDI); BGF, budesonide/glycopyrronium/formoterol fumarate dihydrate; BUD/FORM, budesonide/formoterol fumarate dihydrate (via DPI); CI, confidence interval; COPD, chronic obstructive pulmonary disease; DPI, dry-powder inhaler; FEV_1_, forced expiratory volume in 1 second; GFF, glycopyrronium/formoterol fumarate dihydrate; ICS, inhaled corticosteroid; LABA, long-acting
β_2_-agonist; LAMA, long-acting muscarinic antagonist; LSM, least squares mean; MDI, metered-dose inhaler;
mITT, modified intention-to-treat; SE, standard error; SD, standard deviation.

**Supplementary Table S4**. Moderate/severe COPD exacerbations over 24 weeks: participants on ICS/LABA before screening^a^ by population

|  | **Step up to  ICS/LAMA/LABA** | **Switch to  LAMA/LABA** | **Stay on ICS/LABA** | |
| --- | --- | --- | --- | --- |
|  | **BGF  320/14.4/10 µg** | **GFF  14.4/10 µg** | **BFF  320/10 µg** | **BUD/FORM  400/12 µg** |
| **Overall mITT** | | | | |
| N | 252 | 254 | 107 | 124 |
| Patients with exacerbations, n (%) | 39 (15.5) | 70 (27.6) | 25 (23.4) | 32 (25.8) |
| Events, n | 52 | 96 | 29 | 37 |
| Estimated rate of exacerbations (SE), per year^b,c^ | 0.46 (0.07) | 1.00 (0.13) | 0.63 (0.14) | 0.71 (0.15) |
| **Exacerbations^(No recent + moderate COPD)^ population^d^** | | | | |
| N | 98 | 108 | 40 | 48 |
| Patients with exacerbations, n (%) | 10 (10.2) | 32 (29.6) | 6 (15.0) | 8 (16.7) |
| Events, n | 13 | 40 | 7 | 8 |
| Estimated rate of exacerbations (SE), per year^b,c^ | 0.28 (0.09) | 0.88 (0.17) | 0.39 (0.16) | 0.44 (0.17) |

^a^In the 30 days before screening. ^b^Model-estimated rate. ^c^The rate of exacerbations per year was the total number of exacerbations divided by the total years of exposure across all participants for the treatment. Time during an exacerbation or in the 7 days following an exacerbation was not included in the calculation of exposure. ^d^Participants from the mITT population without a history of exacerbations in the previous 12 months who had moderate COPD (defined as FEV_1_ 50%–80% predicted normal).
BFF, budesonide/formoterol fumarate dihydrate (via MDI); BGF, budesonide/glycopyrronium/formoterol fumarate dihydrate; BUD/FORM, budesonide/formoterol fumarate dihydrate (via DPI); COPD, chronic obstructive pulmonary disease; DPI, dry-powder inhaler; FEV_1_, forced expiratory volume in 1 second;
GFF, glycopyrronium/formoterol fumarate dihydrate; ICS, inhaled corticosteroid; LABA, long-acting β_2_-agonist; LAMA, long-acting muscarinic antagonist; MDI, metered-dose inhaler; mITT, modified intention-to-treat;
SE, standard error.

**Supplemental Figure S1**: Morning pre-dose trough FEV_1_ change from baseline^a^: participants on ICS/LABA before screening^b^


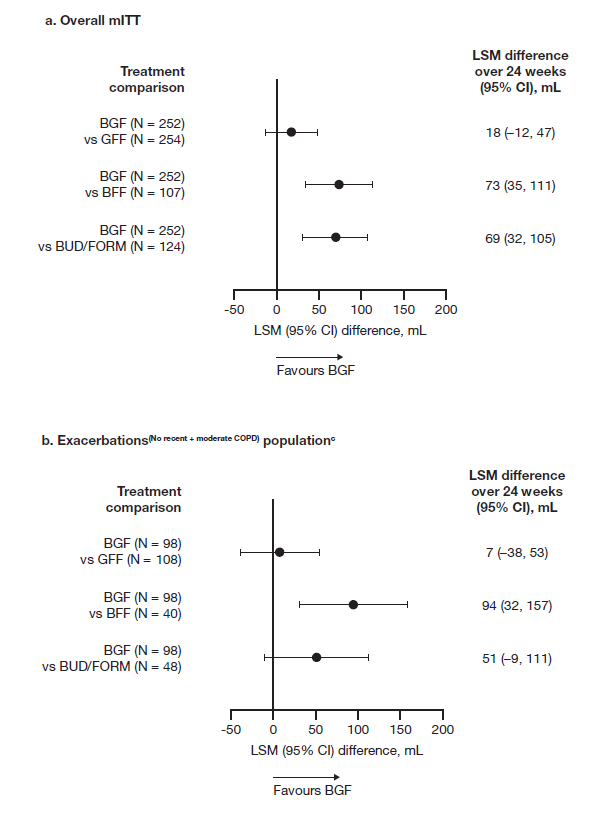


^a^Baseline was defined as the mean of all evaluable 60- and 30-minute pre-dose values on Day 1 (Visit 4) and LSM are presented. ^b^In the 30 days before screening. ^c^Participants from the mITT population without a history of exacerbations in the previous 12 months who had moderate COPD (defined as FEV_1_ 50%–80% predicted normal).

BFF, budesonide/formoterol fumarate dihydrate (via MDI); BGF, budesonide/glycopyrronium/formoterol fumarate dihydrate; BUD/FORM, budesonide/formoterol fumarate dihydrate (via DPI); CI, confidence interval; COPD, chronic obstructive pulmonary disease; DPI, dry-powder inhaler; FEV_1_, forced expiratory volume in 1 second; GFF, glycopyrronium/formoterol fumarate dihydrate; ICS, inhaled corticosteroid; LABA, long-acting
β_2_-agonist; LSM, least squares mean; MDI, metered-dose inhaler; mITT, modified intention-to-treat.

**Supplemental Figure S2**: Moderate/severe COPD exacerbation rate ratios: participants on ICS/LABA before screening^a^


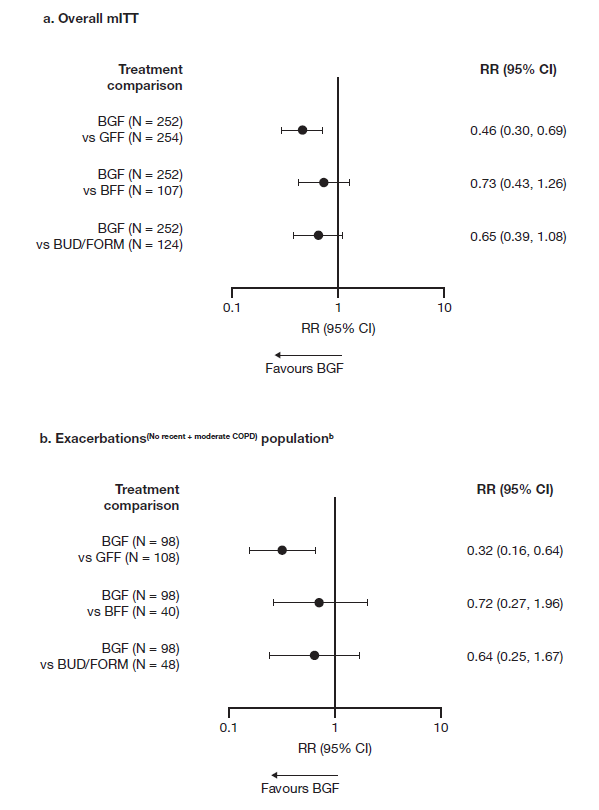


^a^In the 30 days before screening. ^b^Participants from the mITT population without a history of exacerbations in the previous 12 months who had moderate COPD (defined as FEV_1_ 50%–80% predicted normal).

BFF, budesonide/formoterol fumarate dihydrate (via MDI); BGF, budesonide/glycopyrronium/formoterol fumarate dihydrate; BUD/FORM, budesonide/formoterol fumarate dihydrate (via DPI); CI, confidence interval; COPD, chronic obstructive pulmonary disease; DPI, dry-powder inhaler; FEV_1_, forced expiratory volume in 1 second; GFF, glycopyrronium/formoterol fumarate dihydrate; ICS, inhaled corticosteroid; LABA, long-acting
β_2_-agonist; MDI, metered-dose inhaler; mITT, modified intention-to-treat; RR, rate ratio.
