# Supplementary material for: Step up to triple therapy versus switch to dual bronchodilator therapy in patients with COPD on an inhaled corticosteroid/long-acting β2-agonist: post-hoc analyses of KRONOS
Source: Respir Res. 2025 May 8;26:175. doi: 10.1186/s12931-025-03234-5 (PMC12063277; doi:10.1186/s12931-025-03234-5)
Supplement: Supplementary file 2 — Supplementary Material 2: Additional file 2: This document contains a visual summary of the study, including key takeaway points from the background, methods, results and conclusions [file 12931_2025_3234_MOESM2_ESM.pdf]

Step up to triple therapy versus switch to dual bronchodilator therapy in patients with COPD on an inhaled corticosteroid/long-acting β_2-agonist: post-hoc analyses of KRONOS

In people living with COPD receiving ICS/LABA therapy, GOLD recommends a step up to ICS/LAMA/LABA triple therapy for those with no exacerbations but high symptom load ***or*** current exacerbations with blood EOS ≥100 cells/mm[3]

OR

a switch to LAMA/LABA for those with no relevant exacerbation history ***or*** current exacerbations with EOS <100 cells/mm[3]

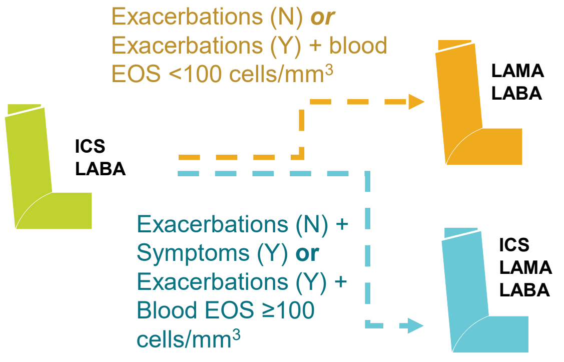

Does a step-up to ICS/LAMA/LABA reduce exacerbation risk vs a switch to LAMA/LABA in symptomatic patients without a recent history of exacerbations?

KRONOS (Phase III, double-blind, randomized study)

BGF	GFF	BFF	BUD/FORM
320/14.4/10 µg MDI	14.4/10 µg MDI	320/10 µg MDI	400/12 µg DPI

An exacerbation history in the past 12 months was not required for KRONOS; **74% of participants in KRONOS had no recent exacerbation history**

ICS/LABA

- Step up to ICS/LAMA/LABA: ICS + LABA + LAMA — **BGF** (N = 188)
- Switch to LAMA/LABA: LABA + LAMA — **GFF** (N = 196)
- Stay on ICS/LABA: ICS + LABA — **BFF** or **BUD/FORM** (N = 170)

24-week treatment period

Post hoc analysis outcomes were assessed among the **74%** **of participants** with no recent exacerbation history

- Lung function (change from baseline in morning pre-dose trough FEV_1) over 24 weeks
- Moderate/severe COPD exacerbation rate over 24 weeks

Supportive analyses were conducted in the overall mITT population (regardless of exacerbation history) and in those with no recent exacerbation history + moderate COPD

Lung function benefit with stepping up to triple therapy with **BGF** was greater versus staying on ICS/LABA with **BFF** or **BUD/FORM**

Exacerbation risk was reduced with stepping up to triple therapy with **BGF** versus switching to LAMA/LABA with **GFF**

Treatment difference (BGF vs dual therapy) over 24 weeks in morning pre-dose trough FEV_1 change from baseline LSM difference (95% CI) mL

BGF vs GFF	BGF vs BFF	BGF vs BUD/FORM
12 (–21, 44) mL	106 mL (64, 148) mL	55 mL (12, 97) mL

Relative reduction in the rate of moderate/severe COPD exacerbations (BGF vs dual therapy) RR (95% CI)

43%	7%	38%
BGF vs GFF	BGF vs BFF	BGF vs BUD/FORM
0.57 (0.35, 0.94)	0.93 (0.47, 1.82)	0.62 (0.33, 1.18)

Supplemental analyses were consistent with findings among participants with no recent exacerbation history

Among KRONOS participants with no recent exacerbation history, stepping up to ICS/LAMA/LABA improved lung function versus remaining on ICS/LABA and reduced exacerbation risk versus switching to LAMA/LAMA.

Despite GOLD recommendations, the potential benefits of stepping up to ICS/LAMA/LABA versus switching to LAMA/LABA, should be considered in patients who remain symptomatic on ICS/LABA.

Abbreviations: BFF, budesonide/formoterol fumarate dihydrate via MDI; **BGF**, budesonide/glycopyrronium/formoterol fumarate dihydrate; **BUD/FORM**, budesonide/formoterol fumarate dihydrate via DPI; **CI**, confidence interval; **COPD**, chronic obstructive pulmonary disease; **DPI**, dry powder inhaler; **EOS**, eosinophil; **FEV_1**, forced expiratory volume in 1 second; **GFF**, glycopyrronium/formoterol fumarate; **GOLD**, Global Initiative for Chronic Obstructive Lung Disease; **ICS**, inhaled corticosteroid; **LABA**, long-acting β_2-agonist; **LAMA**, long-acting muscarinic antagonist; **LSM**, least squares means; **MDI**, metered-dose inhaler; **mITT**, modified intent-to-treat; **RR**, rate ratio.
